# Individual and Organizational Ductility: Conceptualization, Development, and Validation of a New Scale

**DOI:** 10.3390/bs14060511

**Published:** 2024-06-20

**Authors:** Flavio Urbini, Emanuela Caracuzzo, Antonio Chirumbolo, Antonino Callea

**Affiliations:** 1 Department of Humanities, LUMSA University, 00193 Rome, Italy; e.caracuzzo@lumsa.it (E.C.); a.callea@lumsa.it (A.C.); 2 Department of Psychology, Sapienza University of Rome, 00185 Rome, Italy; antonio.chirumbolo@uniroma1.it

**Keywords:** Ductility Scale, validation, measurement invariance

## Abstract

In this article, we conceptualize a new construct named “ductility” and propose a measurement instrument. We examine psychometric properties—the factorial validity and reliability of the Ductility Scale in Italy. The results of exploratory factor analysis showed that the scale has a two-factor structure, namely, individual and organizational ductility. The scale reliability was excellent for both dimensions (individual ω = 0.82; organizational ω = 0.85). The participants were employees from private and public organizations (n = 466). We tested the construct validity of the Ductility Scale. The invariance of the measurement model tested via multigroup confirmative factor analysis showed that the Ductility Scale was invariant across gender. In addition, we found ductility to be positively related to proactive personality and work engagement. These preliminary results show that the Ductility Scale is a reliable and valid measure. In addition, our findings illustrate the potential usefulness of the ductility construct via the newly developed scale.

## 1. Introduction

In the last few years, rapid and unexpected changes have occurred worldwide, such as the COVID-19 pandemic and war conflicts. Referred to as the “pandemic period,” negative consequences on job markets have been well documented by many studies, highlighting the direct repercussions on both social and economic systems [1,2]. Likewise, recent studies showed that the Russia–Ukraine conflict triggered global inflation, reducing the purchasing power of people [3]. According to Chirumbolo and colleagues [4], contemporary society is characterized by high levels of uncertainty felt by individuals in almost every area of daily life. Individuals are forced to manage day-to-day unexpected and sudden changes in their lives, including those related to the work domain. In this regard, some psychological resources like adaptability have become a necessity rather than a choice both within and outside of organizations. Among these psychological resources, here, we propose a new concept called ductility. *Ductility* concerns the ability of an individual to reactively respond to a change requested by the environment. From a cognitive-behavioral perspective, ductility includes the interplay between thoughts, feelings, and behaviors associated with an individual’s response to life or organizational changes.

Being a novelty in the psychological domain, the term still lacks clarification of some of the elements that make it up. In response to these shortcomings, the current study attempted to provide some insight into the concept of ductility and its measurement. Thus, we conducted a preliminary study in Italy to examine a new scale’s factorial validity and reliability, and we assessed the construct validity of this instrument—the Ductility Scale—to assess ductility. Our research aimed to make some important contributions.

First, we introduce ductility as a novel concept within the realm of individual psychological resources, positing that ductility is different from other similar constructs, such as individual adaptability, flexibility, and resilience. Second, we try to explain ductility within the job demands-resources (JD-R) theoretical framework. In our conceptualization of ductility, JD-R theory provides a theoretical perspective for the initiative to face unexpected demands, using one specific personal resource that allow workers to react actively to changes within their work and personal life. JD-R theory helps to explain the potential psychological benefits stemming from being ductile, as well. Third, we developed a new instrument for the assessment of individual and organizational ductility. This short tool can be used in different Italian contexts to assess the psychological resources with which people respond to unexpected demands in daily and organizational life without negative impacts on their mental wellbeing.

### 1.1. From Adaptability to Ductility

The word ductility is commonly linked to a mechanical property. More specifically, in the science of materials, it refers to the “measure of the degree of plastic deformation that has been sustained at fracture” [5] (p. 166). Callister and Rethwisch [5] highlighted the idea that a knowledge of materials’ ductility is important because it provides information about the degree to which the subject might experience a localized deformation without fracture. Considering this, we borrowed the word ductility, thinking that similar dynamics on some level might occur for humans in life and work contexts, as well.

In psychology, the idea of ductility was actually already mentioned in an earlier study. Hobfoll and colleagues [6] used *ductile strength* to refer to the ability of “humans or human systems to undergo change in form (personality or social structure) without breakdown” [6] (p. 175). However, they did not provide a scale with which to measure this *ductile strength*. Therefore, we initially reasoned that the term ductility should be reserved in psychology to describe an individual’s capacity (i.e., a personal resource) to adjust in response to an external pressure. More specifically, in our conceptualization, these pressures come from the environment, that is, we are specifically focused on the personal and working contexts. In this vein, the concept of ductility was inspired by individual adaptability.

*Individual adaptability* has been defined as “an individual’s ability, skill, disposition, willingness, and/or motivation, to change or fit different tasks, social, and environmental features” [7] (p. 13). In fact, an individual with high levels of individual adaptability is able to adapt easily to deal with both obvious changes, such as beginning school, starting a new job, getting married, or becoming partnered as well as minor changes like daily tasks in the workplace. Following Ployart and Bliese [7], individual adaptability is relevant in any situation, not only situations involving obvious change. Extant research has identified a number of positive consequences of individual adaptability, including in the personal domains of self-esteem and life satisfaction and in the work domain in terms of job satisfaction and work engagement [8]. From this point of view, the main difference between individual adaptability and ductility concerns the nature of the concept; ductility is a circumscribed construct compared to adaptability. The major distinction is that adaptability, as a stable individual difference [9], does not occur in response to a specific change in the environment [7], whereas ductility does. In this perspective, ductility is strictly—exclusively—related to the response to an external pressure. In other words, ductility is a response to a characteristic of the situation that is not limited just to a working context, as used in some research on adaptability [10,11]. Let us imagine, for example, that an individual is forced to face an issue that concerns his/her working life (concerning the worry of losing career opportunities) or personal life (concerning becoming a mother or father). Also considering the presence of other main relatively stable individual characteristics (for example, personality traits, skills, and so on) related to the way in which individuals interpret and respond to general situations, ductility is activated exclusively for that specific stimulus—which involves thoughts, feelings, and behaviors—from the environment. In other words, following the examples described above, without the stimulus generated by the worry of losing important job features or that of becoming a parent (i.e., the pressure activated by the environment), ductility does not occur.

Another essential distinction between individual adaptability and ductility can be made regarding the context in which they occur. Although both can be involved in the world of work, adaptability, particularly in the prominent view, is considered only as an antecedent of positive work outcomes, such as task performance [7] and career success [10]; on the other hand, ductility in this preliminary conceptualization has a broader application that does not only refer to how an individual performs on a specific job task. Instead, ductility’s intended meaning is as a personal resource related to a wide range of situations determined by a solicitation from the working environment.

A common issue that individual adaptability and ductility share is activation due to a change in the environment. Following the conceptualization of Ployart and Bliese [7], the analogy with individual adaptability is that ductility can be thought of as *reactive* (when an individual perceives a change in the environment) rather than *proactive* (when an individual perceives a need to change even though the environment has not).

To further substantiate the difference with individual adaptability, it is necessary to specify that ductility is triggered by a specific pressure in the environment. To avoid easy conceptual overlaps, we have to specify that ductility generates only a reactive change—so in a different way than individual adaptability—on a cognitive and behavioral level in a personal and/or work environment.

Moreover, in terms of differences with most conceptualizations and operationalization of adaptability [7,10,11], in our view, ductility includes two different components. The logic behind this distinction is that contemporary society, including the work context, is extremely complex and requires individuals to bring into play some psychological resources at different levels. Based on this, we opted to identify two dimensions of ductility, (1) the first related to the individual’s capacity to react in a flexible and positive way to unexpected changes with innovative approaches, and (2) the second related to the workplace, as the ability to manage unexpected job and organizational changes effectively. The two-dimensional conceptualization adopted to develop the Ductility Scale has the advantage of providing a comprehensive picture of individuals’ psychological resources when facing changes both in general and in the workplace. From a practical point of view, the effects of ductility occur when there is a change in the environment that might be accommodated.

To avoid confusion, it is good to specify that ductility is different from other psychological concepts, such as flexibility and resilience. *Psychological flexibility* refers to the ability to focus on the present moment and, depending upon what the situation affords, persist with or change one’s behavior in the pursuit of goals and values [12]. Therefore, psychological flexibility can be described as the readiness with which the person’s concept system changes selectively in response to appropriate environmental stimuli [13]. Several studies have highlighted how psychological flexibility positively influences the wellbeing of individuals, making them more open to change and able to work effectively [12,14]. In the workplace, more flexible individuals are able to notice opportunities where they can express their valued pattern of behavior, reducing stress and increasing coping strategies [15,16]. What makes this similar to ductility is the reaction to a moment’s change in cognitive-phenomenal terms.

In an analogous way, ductility seems to be similar also to the concept of *resilience*, described as the ability to bounce back from negative emotional experiences by flexibly adapting to changing demands of stressful experiences [17]. From this perspective, resilience can be considered a process or a set of processes, such as capacities or resources deployed in response to adversity, whch allow individuals to withstand ongoing demands and maintain functioning [18]. Furthermore, resilience incorporates the concept of emerging stronger and more resourceful from the adverse situation [19]. Resilience has been associated with a number of positive outcomes. Specifically, a higher level of resilience protects against adverse effects of stress exposure in many life and work contexts [20] and at different stages of life [21]. A common feature of ductility, however, relates to an individual’s capacity to “bounce back” after experiencing stressful life events that cause significant change and contain adverse elements.

What these two constructs have in common with ductility is that they pertain to an individual’s perception of their ability to be flexible or inflexible in a given situation. Although ductility has some commonalities with psychological flexibility and resilience, these terms are essentially different in several aspects. Most notably, ductility is characterized much more by cognitions (i.e., referring to a perceived ability as a cognition) and behaviors rather than just as an effective response, like in resilience. More specifically, ductility is focused on reactively co-changing experiences within one’s own life, not simply bouncing back from stressful events as in resilience, per se. Ductility is different from psychological flexibility in that it is not just a mental strategy but rather a cognitive resource from which specific behaviors spring in reaction to actual changes, also in the workplace, with a functional purpose that facilitates adequate mental functioning. Moreover, contrary to ductility, none of these variables considers the individual’s capacity per their psychological processes that evoke concrete behaviors to reactively respond to daily life changes (e.g., within the work and/or life contexts) in a reactive way requested by the environment. Thus, overall, we deliberately did not refer to similar concepts like resilience and psychological flexibility, as their characteristics are distinct. Rather, we propose that ductility connotes a personal resource with which people respond to changes in daily and organizational life in a reactive way but without negative consequences on their mental wellbeing.

In sum, the novel aspect of the ductility concept that is worthy of highlighting compared to other similar variables is that it can be considered as a personal resource emerging only in response to an environmental pressure related to life or work changes. More specifically, the elements characterizing ductility compared to the variables reported above are the following: it occurs only in response to a pressure in the environment (vs. individual adaptability); it has a cognitive activation (vs. psychological flexibility) followed by a behavioral response (vs. resilience).

### 1.2. Ductility Supported by JD-R Theory

JD-R theory proposes that although working conditions may be quite different for different organizations, all job characteristics can be categorized either as job demands or as job resources [22]. Whereas job demands concern several different job aspects (i.e., physical, psychological, social, and organizational features) that draw on an employee’s efforts or skills, job resources refer to physical, psychological, social, or organizational aspects that are useful in achieving work goals, reducing or managing job demands, and encouraging personal growth. More specifically, according to the health impairment process, when employees feel an unbalanced relationship between job demands and resources, they may eventually voice health complaints. In light of the related empirical evidence and the JD-R framework, ductility may be considered a personal resource that workers can take advantage of and benefit from to counterbalance the aspects implied by job demands (i.e., changes). The current highly unstable work environment negatively and additionally affects several aspects regarding the individual [23]. For example, suppose that an individual has lost a career growth opportunity due the COVID-19 pandemic. From this theoretical perspective, high levels of ductility—considered as personal, i.e., job resources—could permit the individual to react effectively to this job demand, for instance, by trying to increase his/her job performance. From this perspective, ductility can be understood as a protective factor vis-à-vis a worker’s mental health, like, for instance, positive self-evaluations [24]. Furthermore, conceptualizing ductility with JD-R theory can help frame ductility as a motivating resource that is useful in satisfying work objectives, boosting performance and job satisfaction [25]. Indeed, ductility as a job resource might reduce job demands when work pressure is high, helping employees face unexpected changes. Conversely, when work pressure is low, ductility may help employees preserve energy and maintain work interest and motivation.

For example, everyday working demands could represent a stressful event worsening the normal fluctuations in mood in most workers, with a negative consequence towards outcomes related to the job. Ductility as a motivating resource might be useful in managing day-to-day working pressure. For example, suppose an employee in the health sector has to work and devote much time to dealing with diverse needs and requests and problems and complaints from customers and clients (i.e., demands from the environment). A high level of ductility might permit the employee to adapt peacefully to unexpected situations and not give up when managing such working issues.

Therefore, the motivational process of JD-R theory supports the idea that employees with high levels of ductility peacefully adjust to unexpected situations and never back down from organizational changes, positioning ductility as a personal and job resource that is useful in managing job and personal demands.

### 1.3. The Present Study: Development and Validation of the Ductility Scale

Despite our interest in pioneering the ductility concept [6], the lack of a specific scale has limited research on this topic and produced few studies about its relationship to other psychological and organizational variables. To fill this gap, the development of a psychometrically robust and theoretically founded scale is necessary. Based on these considerations, the present research aimed to introduce and validate the Ductility Scale, a new self-report instrument to assess individual and organizational ductility. In what follows, we summarize the phases of the development of such a measure [26].

Firstly, after a thorough review of the literature relevant to similar concepts of ductility, i.e., individual adaptability, flexibility, and resilience, we developed a pool of items. The relevance of items was assessed by a discussion among the authors; no external expert judges were involved, because ductility is a new and little-known concept. The items covered two crucial real-world contexts of a human being’s life: personal and work. In this way, our main goal is to assess the level of ductility in these contexts. This initial evaluation left four items with which to measure each dimension (individual and organizational) of ductility. Regarding the scale characteristics, we have strived to keep the number of items as low as possible to increase the chances that it would be completed, using a self-report measure that seems appropriate both for the measure of individual perceptions and that permits easy administration of the scale in multiple contexts.

Secondly, we explored the scale’s factorial structure in order to investigate whether the results support the theoretical two-dimensional conceptualization. Thirdly, we analyzed measurement invariance across genders and reliability. Finally, we explored the scale’s construct validity, evaluating convergent and discriminant validity among the ductility dimensions, and we sought to theoretically relate psychological variables such as proactive personality and work engagement.

In sum, we conceptualized a new construct named ductility and provided preliminary findings on the development and validation of a short, eight-item tool named the Ductility Scale. Within the realm of the scientific literature on personal resources, ductility could also be a fruitful area for research on these. In this regard, for example, ductility can be used to contextualize how such a concept might impact individual/worker wellbeing.

## 2. Materials and Methods

### 2.1. Procedure and Sample

The present study was part of a research project entitled “Playful work design and flow experience: Antecedents and outcomes”, approved by the Ethics Committee of LUMSA University in Rome. The research protocol consisted of an online questionnaire using Google forms, administrated by snowball sampling. Then, participants were personally contacted via email by three researchers. On the first page of the online questionnaire, we inserted an informed consent notice in which we specified that all participation was free and voluntary, and that the data would be utilized in an aggregated manner. More specifically, the protection of personal data was obtained through total anonymization, which makes it impossible to trace the name of the participant. The choice of this method, which allows for complete confidentiality, derives from the fact that the research has no profiling, diagnostic, or screening purposes. Therefore, the data collected have been processed globally in a password-protected file. This procedure prevents the establishment of any interaction between the participant and the completed questionnaire and consequently does not allow for the return of information to the participant. The sample consisted of 466 Italian employees. The sample was well balanced across gender and education. Regarding age, the highest percentage of participants was aged 26–35 (26.7%), and the other age brackets/intervals were equally balanced. Most of the employees worked in private organizations rather than public organizations, predominantly with a permanent contract. Finally, the average organizational tenure was 10.65 years (SD = 11.12). All sociodemographic and work characteristics are reported in Table 1.

### 2.2. Measures

Our Ductility Scale was developed by conceptualizing items based on the previous literature [6] and our operationalization. Therefore, a pool of eight items was developed for ductility. The authors engaged in discussions to ensure clear and appropriate language and consistency of the content with the primary definition. The items (see Table 2) were formulated to assess individual (four items) and organizational (four items) ductility. The respondents were asked to indicate the extent to which they strongly agreed or disagreed with statements relating to ductility, using a Likert scale ranging from 1 (“strongly disagree”) to 5 (“completely agree”).

“Proactive personality” was assessed using the Italian version [27] of the Proactive Personality Scale (PPS) [28]. This scale measures personal initiative to act with an aim. It is composed of 10 items (sample item: “I am enthusiastic about my job”) on a scale ranging from 1 (“absolutely false”) to 7 (“absolutely true”). In the present study, the McDonald’s omega was 0.90.

“Work engagement” was assessed by the Italian version [29] of the short version of the Utrecht Work Engagement Scale [30]. It assesses the extent to which employees are enthusiastic about and absorbed in their job. The scale was composed of nine items (sample item: “I am enthusiastic about my job”), with a Likert scale ranging from 0 (“never”) to 6 (“always”). In the present study, the McDonald’s omega was 0.94.

### 2.3. Statistical Analyses

Descriptive statistics, item analysis, exploratory factor analysis (EFA), and reliability were assessed using Jamovi software (The jamovi project, 2024) Regarding item analysis, skewness and kurtosis values between −1 and +1 were considered indicative of a normal distribution [31]. For the EFA, we initially used the Kaiser–Meyer–Olkin (KMO) statistic and Bartlett’s test of sphericity. A KMO higher than 0.7 and a statistically significant Bartlett’s test result indicated the appropriateness of the data for factor analysis. The EFA was performed with a principal axis-factoring extraction method, with promax rotation with Kaiser normalization, and using parallel analysis in order to identify the number of factors. Factor loadings higher than 0.30 indicated a good measurement of latent variables [32]. Furthermore, reliability was assessed using the omega coefficient [33], which suggests a good reliability when it is higher than 0.70.

Subsequently, a multigroup confirmatory factor analysis (CFA) was performed using structural equation modeling with Mplus v.8 software. The multigroup CFA aimed to assess measurement invariance across genders, using the maximum likelihood estimator [34]. In line with widely accepted recommendations and guidelines [35], configural, metric, scalar, and structural invariance were tested and compared in that order. The model was evaluated by the following fit indices: χ^2^, comparative fit index (CFI), the Tucker–Lewis index (TLI), the standardized root mean square residual (SRMR), and the root mean square error of approximation (RMSEA). According to literature guidelines [36], CFI and TLI > 0.90 and RMSEA and SRMR < 0.08 suggest an acceptable fit. The comparison of invariance nested models was assessed by Δχ^2^. Specifically, a significant Δχ^2^ suggested rejecting the null hypothesis of invariance.

In order to assess common method variance in a cross-sectional study, Harman’s one-factor test [37] was carried out on items of ductility, proactivity, and work engagement. Specifically, if the total variance extracted by one single factor exceeded 50% means, that common method bias is present in the study. Furthermore, we compared the one-factor model, in which all items were loaded on a single factor, to the hypothesized four-factor model, in which the items were loaded on their expected factor (i.e., individual ductility, organizational ductility, proactivity, and work engagement). A significant Δχ^2^ suggested that the hypothesized model fits better than the one-factor model.

Moreover, descriptive statistics, including skewness and kurtosis, were calculated for the latent variables in order to assess the normal distribution of the sample and allow for parametric statistics (e.g., Pearson’s correlation coefficients). Finally, in order to provide evidence of construct validity, we assessed the correlation between the dimensions of the Ductility Scale, proactive personality, and work engagement. Specifically, we evaluate convergent validity through composite reliability (CR) and average variance extracted (AVE) values and the discriminant validity through maximum shared variance (MSV) analysis. The previous literature [38] suggested that for convergent validity, CR evidence should be higher than 0.7, and the AVE value should be higher than 0.5. Furthermore, per discriminant validity evidence, MSV values should be lower than AVE values [38].

## 3. Results

Preliminary descriptive statistics are reported in Table 2. No item had an extreme mean value, and none had a low standard deviation. Furthermore, skewness and kurtosis values fell between −1 and +1. These results reflected good psychometric properties of the Ductility Scale’s eight items, suggesting a normal distribution.

**Table 2:** Descriptive statistics, item-total correlation, and factor loadings from EFA (promax rotation).

Item	M	SD	Skew.	Kurt.	*ρ_XR_*	λ	
						Ind. Duc.	Org. Duc.
Item 1. I adapt peacefully to unexpected situations [*Mi adatto serenamente alle situazioni impreviste*].	3.53	1.06	−0.39	−0.49	0.63	0.72	0.07
Item 2. I never give up in the face of change [*Non mi scoraggio davanti ai cambiamenti*].	3.70	1.14	−0.64	−0.37	0.66	0.82	−0.08
Item 3. I manage change positively, trying to benefit from it [*Gestisco il cambiamento in modo positivo, cercando di trarne beneficio*].	3.76	1.05	−0.59	−0.27	0.73	0.82	0.01
Item 4. I like to face new situations with innovative approaches [*Mi piace affrontare le nuove situazioni con approcci innovativi*].	3.76	1.04	−0.57	−0.25	0.51	0.33	0.24
Item 5. When the organization promotes a change, I never hold back [*Quando l’organizzazione promuove un cambiamento non mi tiro mai indietro*].	3.76	1.02	−0.54	−0.20	0.63	0.13	0.67
Item 6. When I am entrusted with new work requests, I am able to deal with them effectively [*Quando mi vengono affidate nuove richieste lavorative riesco a fronteggiarle efficacemente*].	3.87	0.89	−0.60	0.34	0.68	−0.08	0.92
Item 7. When the organization implements changes, I am able to manage them calmly [*Quando l’organizzazione attua dei cambiamenti sono in grado di gestirli serenamente*].	3.67	0.99	−0.45	−0.25	0.71	0.16	0.62
Item 8. I have never had a problem showing off my skills in managing organizational change [*Non ho mai avuto problemi a mostrare le mie abilità nel gestire i cambiamenti dell’organizzazione*].	3.72	1.03	−0.50	−0.23	0.70	0.01	0.71

Note. M = mean; SD = standard deviation; Skew. = skewness; Kurt. = kurtosis; *ρ_XR_* = corrected item-total correlation; λ = factor loadings; ind. duc. = individual ductility; org. duc. = organizational ductility. The original Italian items are reported in italics.

**Figure 1:**
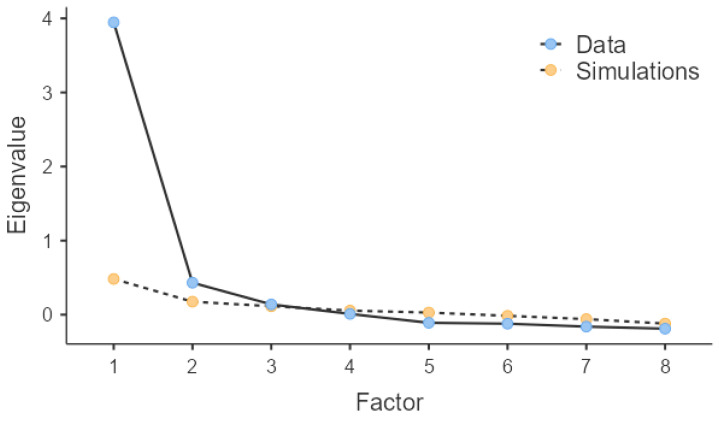
Exploratory factor analysis with parallel analysis.

The EFA results showed the appropriateness of the data for factor analysis, indicating a KMO value of 0.89 and the statistically significant value of Bartlett’s test of 1, 171(28) < 0.001. Parallel analysis suggested the extraction of two factors, as shown in the scree plot (see Figure 1), which explained 57.2% of the total variance. The first factor was composed of four items pertaining to general ductility towards changes and unexpected situations in general life and was therefore denominated “individual ductility.” The second factor was composed of four items concerning the ability to react positively to changes and unexpected situations in organizational life, therefore denominated “organizational ductility.” Furthermore, the factor loadings (Table 2) were between 0.33 and 0.82 for items of individual ductility and between 0.62 and 0.92 for items of organizational ductility. Finally, the two-factor model showed good fit indices: χ^2^(13) = 75.4, *p* < 0.001, TLI = 0.923, RMSEA = 0.081.

Configural invariance across gender showed good fit indices, supporting the equivalence of the configural structure for men and women as reported in Table 3. Furthermore, the Δχ^2^ of the comparing metric and configural invariance models was non-significant, showing that the factor loadings did not load differently across gender. Finally, the significant Δχ^2^ between configural and scalar invariance models suggested a different intercept between men and women (Table 3).

Concerning the reliability of the scale, the McDonald’s omega was 0.82 for individual ductility and 0.85 for organizational ductility, respectively. Furthermore, the item total correlations (see Table 2) ranged from 0.51 to 0.73 for individual ductility and from 0.63 to 0.71 for organizational ductility. Thus, the two dimensions of the Ductility Scale showed good reliability.

Before assessing convergent and discriminant validity of the Ductility Scale, Harman’s test was carried out. The results showed that the total variance extracted by one single factor was 41.1%. Furthermore, fit indices of the hypothesized model, χ^2^(293) = 1020, CFI = 0.91, TLI = 0.90, SRMR = 0.05, and RMSEA 0.07, fit better than those of the one-factor model, χ^2^(324) = 3051, CFI = 0.65, TLI = 0.62, SRMR = 0.10, and RMSEA = 0.13. In fact, the Δχ^2^ = 2031, with Δdf = 31, is significant with *p* < 0.001. These results excluded the presence of common method bias in the present study.

Finally, descriptive statistics of asymmetry and kurtosis (see Table 4) showed that no item violated normality assumptions; therefore, parametric statistics can be used. Pearson’s correlation (see Table 4) analysis showed that both individual and organizational ductility were significantly and positively related to proactive personality and work engagement. Moreover, two ductility dimensions reached good CR (0.73 for individual ductility and 0.76 for organizational ductility, respectively) and AVE (0.54 for individual ductility and 0.58 for organizational ductility, respectively) values. Furthermore, both dimensions had an MSV equal to 0.52 (i.e., the square of correlation between individual and organizational ductility). With CR values higher than the cut-off and AVE values, and AVE values higher than the cut-off and MSV values, these results support the scale’s convergent and discriminant validity.

## 4. Discussion

The present research offers evidence for the validity and reliability of the newly developed Ductility Scale. Compared to instruments that measure similar constructs like flexibility and resilience, this new scale provides an integrated two-dimensional measure of ductility while simultaneously measuring, in a parsimonious and balanced way, a positive cognitive resource at both the personal and work levels.

With data obtained from a sample of employees mainly employed in private organizations on permanent contracts, this study provides evidence for the factorial and construct validity of the Ductility Scale, along with showing the scale’s measurement invariance across genders.

The main contribution of the present research is the refinement of a concept and a new scale for the assessment of ductility. Despite some previous research focused on similar concepts (e.g., individual adaptability, resilience, and psychological flexibility), the refined psychological construct of ductility focuses on specific individual psychological processes followed by behaviors that reactively respond to daily life changes triggered by the environment. The Ductility Scale aims to measure those behaviors that help individuals react in flexible and positive ways to unexpected changes with innovative approaches; preliminarily, the scale also appeared to assess an individual’s ability to manage unexpected job and organizational changes effectively.

For purposes of measurement, we chose to keep the Ductility Scale as short as possible. This shortness is appreciable compared to other major scales that measure similar constructs, i.e., the I-ADAPT-M for individual adaptability [7], with 55 items; the Multidimensional Psychological Flexibility Inventory, [39] with 30 items; and the Connor–Davidson Resilience Scale [40], with 25 items.

Theoretically, ductility can be interchangeable with individual adaptability, because both involve a behavioral change related to environmental stimuli. However, ductility differs from adaptability in that the former involves a reactive change to a personal and/or work environment. Ductility, however, does not involve a proactive way of perceiving the environment but rather refers solely to a reactive way of responding to a specific change in the environment. Therefore, individuals high in adaptability perceive a need to change, even though the environment has yet not changed [7].

Also, the results of our study highlight the idea that ductility is positively related to a proactive personality and work engagement, which provides evidence for the construct validity of ductility. When employees respond to changes in daily and organizational life in a reactive way, they are using a personal resource. Such a ductile approach could have positive consequences on people’s mental wellbeing. Therefore, we argue that ductility might have positive repercussions on individuals’ work and personal lives. Indeed, our results suggest that ductility is positively related both to a proactive personality (i.e., a personal resource) and with work engagement as a work variable. Furthermore, our correlational analyses indicated that ductility is largely related to the elements of a mental health protective factor and motivational resource per JD-R theory (i.e., proactive personality and work engagement). It would be interesting to test both elements with longitudinal designs. It would also be interesting to test whether ductility improves others’ mental health protective factors and motivational resources. In this vein, future longitudinal studies might investigate whether ductility is the antecedent and/or a consequence of other positive individual and work variables.

Although we did not empirically test the comparison with the variables cited above, the results showed a solid factorial structure with an excellent reliability for both dimensions (individual and organizational) of ductility. Based on these findings, it is possible to assume that ductility is a concept that is inspired by individual adaptability but is different from it and other similar constructs.

To our knowledge, no assessment of ductility had been proposed until now. We found that the Ductility Scale had excellent internal consistency/reliability for both dimensions. EFA showed that a two-factor (individual and organizational) solution better fit the data than a one-factor solution.

In the multigroup CFA analyses, evidence for metric invariance was found, which can be considered proof of the strong factorial invariance [41] of the Ductility Scale, meaning that the male individuals perceived ductility similarly to the females. In other words, these results confirmed the statistical equivalence and robustness of the factorial structure per gender.

Thus, adopting a psychometrically rigorous approach, we suggest that future research on ductility in Italy uses our eight-item scale split in two. The internal consistency of the two subscales (individual and organizational) was very high, meeting the stringent criterion of 0.70 for scales in an initial stage of development [42,43].

Finally, researchers have yet to gain a complete understanding of whether individual and organizational ductility have at least a different nomological network (i.e., different causes and consequences). Future research might investigate the possible predictors and outcomes of ductility to expand the nomological network related to the dimensions of ductility. Regarding validity, the data of our study suggest the necessity of further studies to verify other validity aspects, like the predicitive/discriminant validity, of the Ductility Scale.

In practice, one can opt for using the two-dimensional Ductility Scale in a multiple regression analysis and, for example, in testing structural equation models as well, although the present study was not without its limitations. To begin with, non-probabilistic sampling is a limitation, as are the different characteristics within the sample. Despite the sample being well balanced across gender and education, the majority of the participants had a permanent contract and were employed in public organizations. This may limit the generalizability of the findings to other groups of workers. Furthermore, some variables that may influence ductility levels were not included in this study. For example, openness traits or openness to changing may address individual ductility; in the same way, experience in a job position may help employees in the development of organizational ductility. Further studies may investigate the relationship between personal and job variables in the prediction of ductility facets. Moreover, because of the snowballing procedure, the sample cannot be considered as representative of Italian workers. Another issue that also needs to be addressed is that all participants were Italian; thus, it is not possible to generalize our findings regarding the Ductility Scale to other nationalities. Future research should consider more representative samples for specific characteristics and consider other nationalities in order to test the invariance of the Ductility Scale across countries and work categories. Another potential limitation is the self-reported nature of the Ductility Scale; people may not perceive themselves accurately. However, an individual’s perception of ductility is highly subjective, and a self-report measure seems more than appropriate. Another limitation regards the concept of ductility. For instance, it remains unknown how ductility is related to similar concepts, such as individual adaptability, resilience, and psychological flexibility. Although we reasoned earlier that ductility differs from the cited concepts, future research is needed to ascertain the discriminant validity of ductility from these and other comparable variables.

These findings can be considered as a starting point for future studies to consider this personal resource more in depth. The availability of a brief and simple scale about the measure of ductility may have some important implications both for individuals and organizations. From an individual point of view, the Ductility Scale may be used in order to assess the personal capacity to react to unexpected changes in personal life with a positive approach. This information may help people to better understand themselves and to choose a personal development plan in order to improve this ability. From an organizational point of view, the shortness of the Ductility Scale allows for easy and quick use, together with other scales, in several organizational processes. For example, measuring ductility in the selection phase may help recruiters to assess candidates for roles requiring turbulent or changing activities. In skills assessment, ductility may provide important information to HRMs about the ability of employees to acquire new roles or new competences or to attend to skill training. Finally, in line with the JD-R model [22], job and organizational demands request physical, psychological, social, and organizational effort from employees; therefore, ductility may be a key personal resource that helps employees to better respond to organizational changes and demands. Therefore, learning about and improving ductility may be a crucial strategy for individuals who seek positive outcomes. By stimulating individual and organizational ductility, individuals may be able to face higher levels of uncertainty that are felt in almost every area of daily life these days. For example, in the context of work, competitive organizations might help improve employees’ psychological resources like ductility to enable their workers to manage day-to-day unexpected changes.

## 5. Conclusions

In this research, we developed and validated a new instrument for the measurement of ductility. The Ductility Scale had adequate psychometric properties, showing encouraging results on the construct’s reliability and factorial/construct validity. Ductility was positively related to a proactive personality and work engagement, resulting in a distinct concept. These findings offer evidence for the construct validity of the scale.

Overall, the present study has introduced a self-report scale for assessing two major dimensions of ductility that might be adopted usefully, both for research and practice in Italy. Therefore, we hope this research stimulates studies on the positive personal resource of ductility, which can be used within and outside of organizations.

## Figures and Tables

**Table 1:** Sociodemographic and work characteristics of the sample (n = 466).

Characteristics		n	%
Gender	Males	231	49.7
	Females	234	50.3
	Missing	1	
Age intervals	18–25	79	17.0
	26–35	124	26.7
	36–45	81	17.5
	46–55	90	19.4
	>55	90	19.4
	Missing	2	
Education	Compulsory education	196	42.2
	University degree	269	57.8
	Missing	1	
Contract type	Temporary employees	118	25.4
	Permanent employees	347	74.6
	Missing	1	
Organizational sector	Private organizations	149	33.3
	Public organizations	299	66.7
	Missing	18	

**Table 3:** Results of invariance across gender (males: n = 231; females: n = 234).

Invariance	χ^2^	*Df*	CFI	TLI	SRMR	RMSEA	Δχ^2^	Δ*df*	*p*
Configural invariance	139.32	62	0.986	0.987	0.038	0.073	-	-	-
Metric invariance	142.36	68	0.986	0.989	0.038	0.069	3.04	6	ns
Scalar invariance	184.07	78	0.978	0.982	0.037	0.088	41.71	10	<0.001

**Table 4:** Pearson’s correlation, CR, AVE, and MSV.

	1	2	3	4	CR	AVE	MSV	M	SD	Skew.	Kurt.
1. Individual ductility	0.82	0.72	0.58	0.42	0.73	0.54	0.52	3.69	0.86	−0.53	−0.18
2. Organizational ductility		0.85	0.72	0.58	0.76	0.58	0.52	3.75	0.81	−0.49	0.24
3. Proactive personality			0.90	0.63				3.70	0.73	−0.71	0.75
4. Work engagement				0.94				3.63	0.87	−0.54	−0.13

Note. CR = Composite reliability; AVE = average variance extracted; MSV = maximum shared variance (MSV); M = mean; SD = standard deviation; Skew. = skewness; Kurt. = kurtosis.

## Data Availability

The data used in this study are available on request.
